# A plain abdominal x-ray may direct the diagnosis of primary hyperoxaluria

**DOI:** 10.1590/2175-8239-JBN-2023-0032en

**Published:** 2024-02-12

**Authors:** Maria Helena Vaisbich, Diane Xavier de Ávila, Romulo Cézar Pizzolatti

**Affiliations:** 1 Universidade de São Paulo, Faculdade de Medicina, Hospital das Clínicas, São Paulo, SP, Brazil.; 2 Complexo Hospitalar de Niterói, Rio de Janeiro, RJ, Brazil.; 3 Clínica de Nefrologia de Araranguá, Araranguá, SC, Brazil.

**Figure 1:**
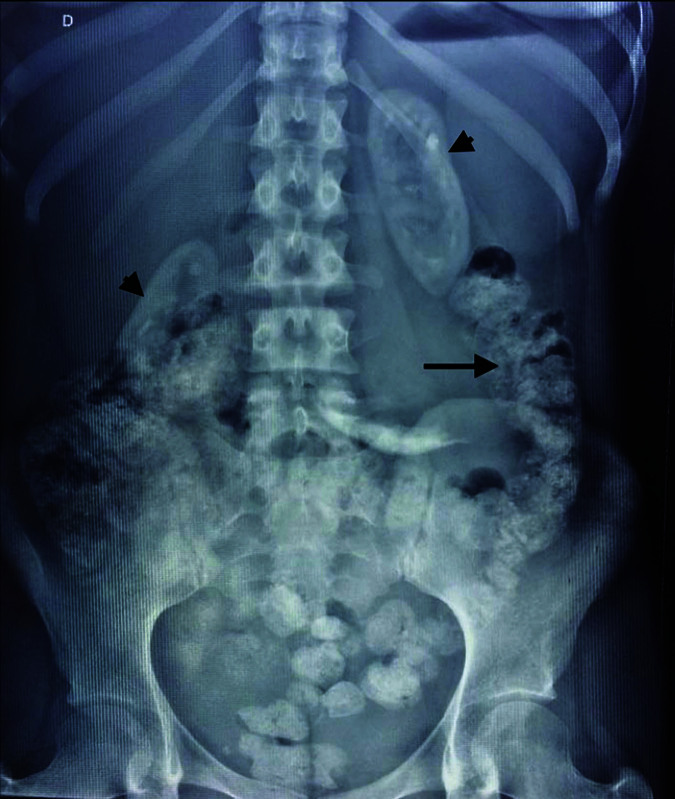
Plain abdominal x-ray showing colonic calcifications (arrow) and nephrocalcinosis (arrow head) in a patient diagnosed with primary hyperoxaluria type 1.

**Figure 2:**
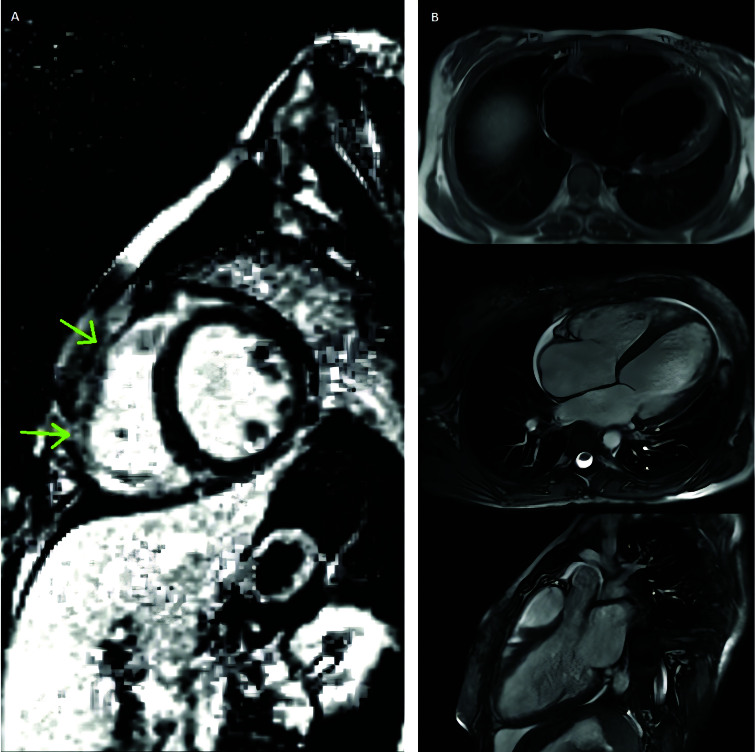
A. Cardiac MRI showed dilated cardiomyopathy with significant impairment of global left and right ventricle function. B. Delayed enhancement revealed linear mesocardial fibrosis in the septal region (arrows).

This is a case report of a 23-year-old female patient on hemodialysis for 1.5 years due to nephrocalcinosis and recurrent kidney stones. A plain abdominal x-ray showed nephrocalcinosis and extrarenal involvement such as bone disease and an unusual location of crystals in the digestive tract characterized by calcifications in the colonic wall (Figure 1). This patient also presented a suggestive cardiac involvement of the disease (Figure 2). Plasma oxalate levels were high and genetic testing revealed primary hyperoxaluria type 1^1^. The patient had heterozygous compound mutations in *AGXT* previously described (- allele 1: c.508G>A;p.Gly170Arg – allele 2: c.661_663delTCC;p.Ser221del)^1,2^. This diagnosis is very important for both hemodialysis planning and renal transplant strategy. A literature review shows that 3 cases of digestive tract deposits identified by abdominal ultrasound or computed tomography have been reported to date^3^. Importantly, there are new treatment options available for this disease^4^.
